# Responsible north–south research and innovation: A framework for transdisciplinary research leadership and management

**DOI:** 10.1016/j.respol.2024.105048

**Published:** 2024-09

**Authors:** Matthew A. French, S. Fiona Barker, Rebekah Henry, Amelia Turagabeci, Ancha Ansariadi, Autiko Tela, Diego Ramirez-Lovering, Fitriyanty Awaluddin, Ihsan Latief, Isoa Vakarewa, Ruzka R. Taruc, Tony Wong, Brett Davis, Rebekah Brown, Karin Leder, for the RISE consortium

**Affiliations:** a Faculty of Art, Design and Architecture, Monash University, Victoria, Australia; b Faculty of Medicine, Nursing and Health Sciences, School of Public Health and Preventive Medicine, Monash University, Melbourne Australia; c Department of Public Health and Primary Care, Fiji National University, Suva, Fiji; d Department of Epidemiology, School of Public Health, Universitas Hasanuddin, Makassar, Indonesia; e Revitalising Informal Settlements and their Environments (RISE), Makassar, Indonesia; f Department of Engineering, Universitas Hasanuddin, Makassar, Indonesia; g Revitalising Informal Settlements and their Environments (RISE), Suva, Fiji; h Monash Sustainable Development Institute, Monash University, Victoria, Australia; i Office of the Deputy Vice Chancellor and Senior Vice President, Monash University, Victoria, Australia; j RISE consortium (https://doi.org/10.26180/ctjf-vf69)

**Keywords:** Research management, Transdisciplinary, Sustainability, Global south, Research leadership, Decolonising research

## Abstract

The number, scale and ambition of transdisciplinary research initiatives between the global north and the global south is increasing, yet there is very little theoretical or empirical scholarship on how to lead and manage implementation to promote responsible practice. Within science, technology and innovation (STI) studies and decolonising research frameworks, and utilising collaborative autoethnography, this study codifies experience with implementing the ‘Revitalising Informal Settlements and their Environments’ (RISE) program (2017–2020). Our specific aim is to explore the leadership and management tensions and challenges of implementing transboundary transdisciplinary research. The findings reaffirm the importance of research leaders and managers carefully operationalising north–south research by critically reflecting on power asymmetries between disciplines, partners and locations, leveraging the potential for transdisciplinary consortia to build research capabilities in the global south, and creating a culture of reflexivity on the historical and social positionality in which research is designed, funded, implemented and evaluated. The findings foreground the role of boundary-spanning ‘integrators’ and ‘pracademics’, roles that have received little attention to date but are essential for effective delivery and societal impact beyond scientific advances. A framework for implementing north–south transdisciplinary research is outlined with five domains: (1) collaborative leadership; (2) agile management; (3) flexible consortia; (4) researcher positionality; and (5) co-design and participation. The framework can support efforts for responsibly designing and implementing large, transdisciplinary, cross-country research programs in line with ambitions for decolonising north–south research.

## Highlights

• Implementing large-scale north–south transdisciplinary research for socio-technical innovation is challenging.
• We present a case study focusing on implementation tensions and challenges of leadership and management.
• Boundary-spanning ‘pracademics’ and ‘integrators’ are essential for effective implementation.
• We outline a framework for implementing innovative research using a responsible decolonised north–south research praxis.

## Graphical abstract

**Figure:**
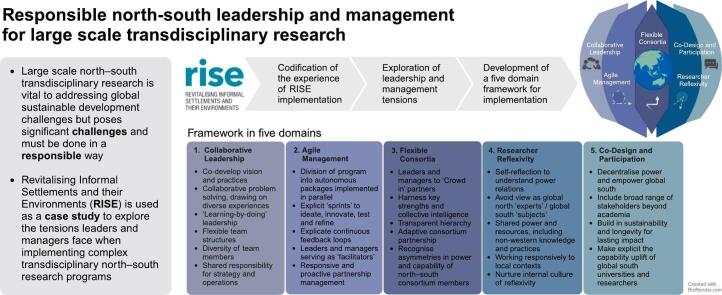

## 1 Introduction

### 1.1 Transboundary transdisciplinary research for SDG innovation

Solving global sustainable development challenges requires transformational scientific research systems that provide rigorous evidence through integrated, innovative solutions and strong, cross-sector and cross-country partnerships (Fazey et al., 2020). Global coordination is central to achieving the Sustainable Development Goals (SDGs), with Goal 17 articulating principles and actions to deepen partnerships for innovation, including better linkage of universities and research institutes with policy makers and program implementers (Brennan and Rondón-Sulbarán, 2019; Zingerli, 2010; Scholz, 2020).

The United Nations has called on all sectors to accelerate global research and innovation to meet the SDGs by 2030 (UN, 2021). This requires research that is transdisciplinary: research co-designed and co-implemented across traditional academic disciplines as well as with non-academic actors such as communities, governments and non-profit sectors (Hirsch Hadorn et al., 2008; Brown et al., 2010; Carayannis and Campbell, 2009; Peris-Ortiz et al., 2016). The concept of a ‘triple helix’ model involves entrepreneurial co-creation and delivery of research across three sectors: universities, government and industry; the ‘quadruple helix’ model extends this by adding society (public participation and engagement) (Carayannis and Campbell, 2009; Etzkowitz et al., 2000; Godin and Gingras, 2000; Peris-Ortiz et al., 2016). These models have received significant attention in high-income countries (the ‘global north’) (Jahn et al., 2021; König et al., 2013; Hoffmann et al., 2017), however the application of these models in low- and middle-income countries (the ‘global south’) and across north–south contexts remains nascent (Chen et al., 2019; Lang et al., 2012). (See ‘Supplementary material 1’ for an explanation of our use of global north–south terms.)

Implementing north–south transdisciplinary research involves unique and significant challenges and tensions. ‘Helicopter research’ – where global north researchers undertake research in the global south with insufficient engagement of local researchers and local research ecosystems (Erondu et al., 2021; Nature, 2022) – is highly problematic. Key challenges include: significant power asymmetries; research funding differentials (Haelewaters et al., 2021); ethical and moral issues, especially with trials in vulnerable communities where control groups do not receive commensurate or timely benefit (de Souza and Eyal, 2019); cultural differences (Kotze and Dymitrow, 2021); ‘ethics dumping’ as a result of weak research governance, to the detriment of vulnerable populations (Schroeder et al., 2020); and equitable publication or commercialisation of research findings (Etzkowitz, 2003). Transdisciplinary research – which involves non-academic actors in problem identification, research design, implementation, interpretation, dissemination and use of research findings – further exacerbates these challenges (Lang et al., 2012; Scholz, 2020), both because of potential tensions between academics and practitioners, and because of different viewpoints between disciplines and cultural understandings of specific contexts.

There is limited scholarship and guidance on how to effectively lead and manage responsible north–south transdisciplinary research initiatives (Kotze and Dymitrow, 2021; Zingerli, 2010). Empirical studies of transdisciplinary research projects are rare; most are interdisciplinary. Practical guidance remains at a high level, and the latest scholarship and praxis on decolonising global research is not well incorporated.

This paper draws from science and technology studies and decolonising research frameworks to explore the tensions and challenges of leading and managing the ‘Revitalising Informal Settlements and their Environments’ (RISE) program (Brown et al., 2018). RISE is a AU$60 m, 10-year randomised controlled trial being implemented in Fiji and Indonesia involving over 10 disciplines and 28 partner organisations. RISE is testing the human health and environmental impacts of a decentralised, nature-based approach to water and sanitation servicing in urban informal settlements (Leder et al., 2021). (See ‘Supplementary information 2’ for additional background on RISE.)

Our aim is to explore the leadership and management tensions and challenges of implementing transboundary transdisciplinary research. Our three objectives are to: (1) codify the experience of implementing RISE during its first three years (2017–2020), (2) contribute to scholarship on responsible north–south research and innovation; and (3) provide practical guidance for others embarking on or facing challenges implementing similar research programs. We ask: What are the tensions and challenges facing leaders and managers implementing complex transdisciplinary north–south research, and how can these be navigated and reconciled with efforts to promote responsible north–south research?

## 2 Conceptual framework: leading and managing north–south transdisciplinary research

### 2.1 Transdisciplinary research leadership and management

Scholarship on effective implementation of transdisciplinary research points to four key factors mediating success. First are internal factors, including building and maintaining cohesive teams, sustaining trust and constructive dialogue, and aligning actors around a shared vision and goals (Hollaender et al., 2008; Adler et al., 2009; Scholz and Steiner, 2015). Second are external factors, including sufficient funding and flexibility in funding modalities (Campbell et al., 2015; Felt et al., 2016). Third are process factors, including mutual and purposeful learning and adaptation (Hoffmann et al., 2022a); appropriate communication channels for different audiences and different research stages; sufficient timelines; and continued commitment of researchers (Roux et al., 2010; Pohl, 2005); and sufficient flexibility in research design to accommodate changes and incorporate lessons learned during implementation (Senge, 1990; Hoffmann et al., 2022a). Fourth are partnership and sustainability factors, including co-designing the research agenda, making explicit the responsibilities and accountabilities of partners, pooling profits and sharing risks, and sharing and disseminating data and outcomes (Hirsch Hadorn et al., 2008; Scholz, 2020).

Common to all four factors is the fundamental role of research program leaders and managers (Gordon et al., 2019; Vienni-Baptista and Thompson Klein, 2022). Management theory identifies leadership and management as related concepts, but they are also distinct (Yukl, 1989). Leadership refers to the ability of an individual and/or team to set a vision, shape the culture, and inspire and motivate others to accomplish something. Management refers to executing the vision, monitoring performance and delivering activities (Yukl, 1989). Leaders look to and attempt to shape the future; managers work in the present and drive execution.

While individual and organisational leadership and management roles may overlap, the leadership roles in transdisciplinary research programs are typically held by principal and chief investigators (P-CI, CIs) and discipline/work package leads, whereas management roles are typically held by project/program managers and field/country research managers (Hollaender et al., 2008). Depending on program size, there may also be technical managers, data managers and partnership/stakeholder managers (Hollaender et al., 2008; Hansson and Mønsted, 2008).

Effective leaders and managers of transdisciplinary research projects should have experience and tacit knowledge (Clark and Stankey, 2006), should be comfortable with uncertainty, and should demonstrate nuanced capability for agile adaptation to cope with complex systems (Roux et al., 2010). They should sensitively forge a common vision to align disciplines and teams, and nurture ‘t-shaped’ researchers who have both disciplinary depth and transdisciplinary breadth (Brown et al., 2019). They must sensitively steward multiple stakeholders across diverse sectors, often with varied and conflicting expectations, motivations and timeframes (Ernø-Kjølhede, 2001). They must be able to proactively identify, manage and resolve conflict, and reduce the likelihood it escalates *(*Hollaender et al., 2008). They must also bridge disciplines (Adler et al., 2009; Pohl, 2005) and play an essential role in mediating, synthesising and integrating diverse disciplinary methodologies and datasets (König et al., 2013; Hoffmann et al., 2022a).

Transdisciplinary research funding structures are often complex, with consortia (multi-donor) funding increasingly common, often requiring supplemental in-kind contributions that are not necessarily under the direct control of leaders and managers. Matrix organisational structures predominate, rather than traditional academic reporting and accountability hierarchies, often making direct command-and-control impossible (Pohl, 2005). Given all these complexities, there are calls for more humanistic, empathetic research leadership and management given the high social and personal costs to researchers in transdisciplinary projects and tendency for burn-out (Sellberg et al., 2021; Felt et al., 2013).

### 2.2 Responsible north–south research

Implementing transdisciplinary research across north–south countries brings distinctive challenges and tensions. The historical legacy of colonialism has resulted in highly unequal global scientific research ecosystems that disproportionately advantage global north researchers and institutions. Research funding is skewed to institutions and researchers in the global north, and power asymmetries often constrain ethical and effective collaboration (Erondu et al., 2021; Wardani et al., 2022) resulting in explicit or implicit disparities in decision-making authority on research design, resource allocation, division of labour and research outputs (Haelewaters et al., 2021; Nature, 2022). The challenges are exacerbated when non-academic actors such as study communities and local government authorities are engaged in the research, often bringing divergent cultures, expectations, priorities and timeframes. ‘Decolonising’ north–south research initiatives requires nuanced leadership to co-design and co-implement initiatives that recognise and account for these traditional and structural impediments (Lang et al., 2012; Turner et al., 2024).

A range of frameworks and normative guidance attempt to foster more equitability in north–south partnerships. Voller et al. (2021) reviewed 22 guidelines published between 1994 and 2021, finding most are from the fields of international development and global health. The most widely used guide is the ‘*Guide for Transboundary Research Partnerships: 11 Principles*^1^ produced by the Swiss Commission for Research Partnerships with Developing Countries in 1998 and updated in 2012 (Stöckli et al., 2018). The Swiss Guide is acquiescent with two seminal global normative frameworks, the ‘*Montreal statement on research integrity in cross-boundary research collaborations'* (WCRI, 2013) and the ‘*Global Code of Conduct for Research in Resource-Poor Settings'*, a framework of 23 Articles under four value areas: Fairness, Respect, Care and Honesty (Trust, 2018). These frameworks implicitly reflect the crucial role of leaders and managers as boundary spanners between different contexts, cultures, sectors and disciplines. However, the ‘*Guide for Transboundary Research Partnerships: 11 Principles'* remains abstract and aspirational (Kotze and Dymitrow, 2021), providing little guidance about the everyday tactics, systems and tools for leading and managing complex SDG north–south research endeavours. Additionally, it is largely silent on the leadership and management tensions of responsible international research collaborations.

In the field of global health, Larkan et al.'s (2016) framework identifies leadership as a core pillar of effective north–south partnerships, yet the authors conclude that “… leadership dimensions are underexplored in the literature compared to other aspects such as research co-design and dissemination of results”. Newman et al.'s (2019) framework draws from the international development field and outlines eight principles for fair and equitable research partnerships, calling for greater involvement of southern researchers in shaping northern research funding and decision-making.

Outside the field of global health, frameworks such as the *Nagoya Protocol on Access and Benefit-sharing* recognise the sovereign rights of countries to establish specific legislation to support bilateral negotiation of benefits relating to non-human derived genetic material (Smith et al., 2017), thereby fostering fair and equitable sharing of resources for understanding global biological diversity (Buck and Hamilton, 2011). However, challenges remain due to the lack of an overarching legal framework and difficulties developing and communicating country-specific regulations. These factors may result in unexpected barriers for researchers and a lack of clarity to establish successful long-term collaborations (Hoelmer et al., 2023).

### 2.3 What makes an effective leader and manager of north–south research?

Empirical studies that have codified experience provide further evidence on how leaders and managers can navigate complex implementation conditions. Implementing a public health randomised controlled trial in urban slums in India, Mahapatra et al. (2016) found a leader's capability to build deep trust and respect with local counterparts and communities is crucial, yet this takes time and resources, which are often insufficiently planned for and funded.

Similarly, drawing from a sustainable water research collaboration between German, Botswanan, Angolan and Namibian researchers, Schmidt and Pröpper (2017) found one of the most important roles of leaders and managers was managing the tension between the disparate timelines and expectations of the research project and the study communities. The authors further highlight the leadership challenge of enrolling (and maintaining over the life of the program) stakeholders who are not interested in a *research* project, especially government ‘partners’ for whom research permissions are often needed and where ambitions for research translation and scale-up often sit.

Kotze and Dymitrow's (2021, p.15) evaluation of a 10-year north–south research platform and doctoral research capability initiative led out of Sweden with a number of global south countries found “… this failure [of the program] cannot just be laid at the feet of the administrative and institutional structures, but also of the individual actors involved”.

The increasing operational scale and complexity of large transboundary SDG initiatives calls for advanced project management capabilities to ensure timely delivery (Cundill et al., 2018; Saric et al., 2019). Cundill et al.'s (2018, p.1) experience implementing a seven-year climate change adaptation research program involving more than 450 researchers and practitioners across 17 countries found the human relational aspects are key for success, “including interpersonal trust, mutual respect and leadership styles.” The authors identify key enablers of effective leadership and management, including (i) dedicated project coordinators, at multiple levels; (ii) an inclusive and hands-on approach, and (iii) forging friendships, not purely professional relationships. The authors articulate an important role: that of ‘consortium coordinators' *“*… who coordinate rather than do the research … Effective coordinators go beyond traditional management roles, mediating between institutions with different values, being attentive to power asymmetries, and working to navigate systemic barriers such as those embodied in grant agreements*”* (Cundill et al., 2018).

Overall, there is considerable scholarship and guidance about how north–south research collaborations *should* be set up and how they *should* operate, yet limited information on *how* to implement them – particularly for large-scale, multi-country transdisciplinary research initiatives (Voller et al., 2021; Lawrence, 2015). Further, there is limited scholarship and evidence on the roles of research program leaders and managers in implementation: their capacity and capability requirements and the organisational systems and supports for effective delivery of complex, multi-partner transdisciplinary north–south research projects and programs (Zingerli, 2010).

## 3 Methods

### 3.1 Examining innovation processes and practices

This study is guided by science, technology and innovation studies that seek to uncover patterns and processes shaping knowledge production and innovation by examining the consequences of everyday practices on different groups of people (Latour and Woolgar, 1979). Similarly, post-colonial frameworks (Styhre, 2008; Smith, 2012) provide insights into how the positionality of people and hegemonies of knowledge production impact scientific and research endeavours (Harding, 2004; Haraway, 1988). These frameworks support critical reflection on power asymmetries by looking at everyday practices, and the role of people in positions of power (i.e. leaders and managers) in shaping socio-technical outcomes. Our method aligns with grounded theory (Glaser and Strauss, 1967; Creswell, 2013) and implementation science (Eccles and Mittman, 2006). These frameworks enable theory building from empirical evidence and incorporate diverse voices in shaping ‘what works, and why’. We use a case study methodology, which enables exploration even when the “boundaries between the phenomenon and the context are not clearly evident” (Yin, 1984, p.23).

Collaborative autoethnography (CA) is our primary method, underpinned by a self-reflexive praxis approach (Grant and Thompson, 2018). Autoethnography combines autobiography (reflecting and writing about personal experience) and ethnography (the systematic study of individual cultures, explored from the viewpoint of those in it) (Jones et al., 2013). *Collaborative* autoethnography (CA) incorporates both individual and group perspectives, bringing together people who share a common concern, interest or experience to systematically analyse and understand cultural experience (Chang, 2013). The study uses a participative ‘structured reflection’ method (Roux et al., 2010). Working iteratively, we combined our experiences with project data and reports. We co-analysed individual and collective experiences and co-constructed narratives that illustrated our relational experiences as a writing team, the broader internal team and external stakeholders. We framed and tested these accounts against scholarship, normative guidelines and other empirical cases.

The study, conducted between August 2021 and February 2023, involved six main steps: (i) Scoping the study parameters, setting draft aims and objectives, and inviting RISE researchers to be co-authors; (ii) co-author group ‘sensing’ workshops involving the lead author and co-authors, refining the study scope, reflecting on personal experience, and surfacing, discussing and collating inputs for the case study analysis; (iii) self-reflexive writing and discussions which included critical reflection on our positionality and roles in RISE, critically reflecting on how these may influence the emerging findings; (iv) desktop review of RISE project documents (i.e. work plans, reports, minutes of meetings) to fact check, triangulate with our personal accounts, and contextualise our accounts within project documentation; (v) iterative co-author review meetings, workshops and conversations where we discussed and analysed the draft paper, tested and iterating key findings (similarities, differences) and co-developed the framework and five domains; and (vi) providing the opportunity for RISE senior leadership members who are not co-authors to review and incorporation of their inputs to reach a final version of the Framework and paper.

We critically reflect on our own roles and experiences in RISE and highlight how these experiences may have (or not) influenced the analysis and conclusions. As outlined in ‘Supplementary material 3’, the analysis and findings reflect our subjective experiences, our roles in the RISE program (formal and informal, with varying levels of responsibility), and motivation from the outset to present a balanced analysis (i.e. to critically analyse both successes and failures). Furthermore, our positionality within the author group, and within the RISE program, may have influenced the analysis and conclusions, for example power asymmetries across diverse geographies (Australia, Indonesia, Fiji), age and seniority (senior professors, early-career researchers, fieldworkers and lab technicians), perceived value of expertise domains (i.e. academic, project management, community development), and employment type (tenured academic, fixed-term contract). We were the core group of leaders and managers of RISE during the first three years which brings deep experiential knowledge but also potential subjectivity and biases by not including a broader range of experiences, including that of external partners. These aspects required a rigorous and critical self-reflection process to enable critical analysis of our own performance and recognise the limitations of the method, noting that the study is not an evaluation and was not structured according to previously specified criteria. It is retrospective and the outputs (i.e. this paper) should be seen as historically situated.

### 3.2 Case study: Revitalising Informal Settlements and their Environments (RISE)

The RISE program is a 10-year, AU$60 m, transdisciplinary, global north–south research program. The program responds to the public health and environmental challenges of urban informal settlements, home to more than 1 billion people (Brown et al., 2018; Wong et al., 2018). It aims to develop pioneering transdisciplinary evidence on the planetary health links between human health and wellbeing and the environment in informal settlements to upgrade policies and investments to help meet SDG6 (clean water and sanitation) and SDG11 (sustainable cities and communities) (Brown et al., 2018).

RISE comprises a randomised controlled trial in 24 informal settlements: 12 in Suva, Fiji, and 12 in Makassar, Indonesia (Leder et al., 2021). The trial is testing a ‘water sensitive cities’ approach (French et al., 2021a) that includes co-designed (Prescott et al., 2021), nature-based, decentralised water and sanitation technologies (Burge et al., 2021). The intervention theory of change is that by co-designing and implementing settlement-wide decentralised water and sanitation systems, flood protection and access improvements, environmental contamination and resident's exposure to faecal waste will be reduced, which will in turn positively improve environmental and ecological diversity and human health and wellbeing, particularly by reducing the entero-pathogen burden on children (See: French et al., 2021b for the conceptual model underpinning the intervention).

RISE involves 5 work packages (‘Objectives’):

• Objective 1: Develop novel urban design, architecture and infrastructure models in partnership with local communities.
• Objective 2: Assess the impact on environmental contamination and ecology.
• Objective 3: Assess the impacts on human health.
• Objective 4: Assess the broader impacts on residents' wellbeing.
• Objective 5: Facilitate evidence-to-policy and investments.

RISE is a unique case study, with four particularly notable features that distinguish it from other north–south programs: (i) the design and delivery of a highly complex civil engineering intervention in 24 vulnerable communities, (ii) the randomised controlled trial (RCT) research design, (iii) the high degree of transdisciplinarity, and (iv) our newness as a consortium operating in these locations. These dimensions, along with the number of partners and multi-country implementation, created significant demands on all individuals and organisations involved, particularly RISE leaders and managers.

**Fig. 1:**
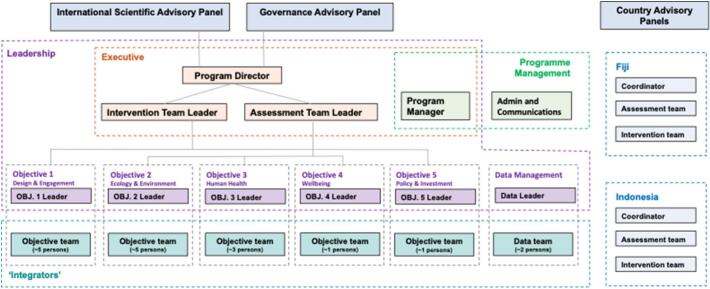
RISE internal organisational structure, 2017–2020. Source: RISE program.

**Fig. 2:**
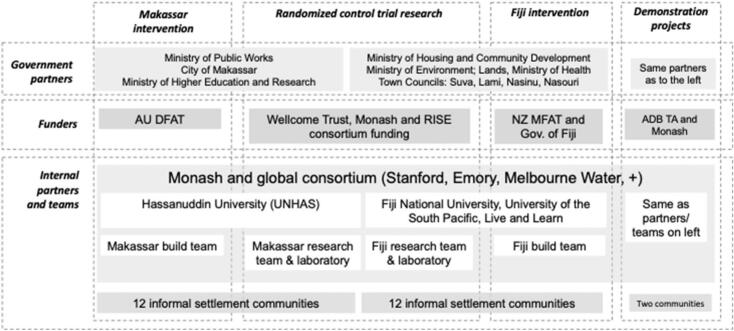
RISE ecosystem showing constituent partners, funding, and locations. Source: RISE program.

RISE was designed and set up with a decentralised, matrix organisational structure (Fig. 1). The Executive group comprised the P-CI, the Assessment Lead, the Intervention Lead and the Program Manager. The Leadership group comprised the five Objective Leads, Data Manager and the Executive members. Each Objective has its own team (mostly early career researchers) who develops and implement their area of research. The Program Manager established and manages teams and operations in Fiji and Indonesia and promotes cross-Objective coordination. Fig. 2 outlines the broader RISE ecosystem showing constituent partners, funding and locations.

### 3.3 Operationalising RISE: June 2017–July 2020

**Fig. 3:**
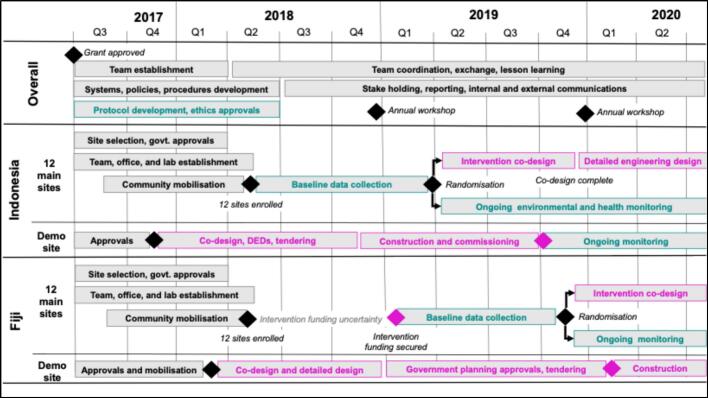
Timeline of RISE, Years 1–3. Source: RISE program. Black text: Whole-of-program activities; Pink: Intervention activities; Teal: Assessment/research activities. (For interpretation of the references to colour in this figure legend, the reader is referred to the web version of this article.)

Year 1 (June 2017–May 2018) focused on setting up the program (Fig. 3). Key tasks involved: (i) establishing operational systems, communications and coordinating structures; (ii) establishing the Fiji and Indonesian teams and research and delivery platforms; (iii) formalising stakeholder partnerships including with government agencies in Fiji and Indonesia; (iv) selecting study sites, obtaining government approvals and mobilising the community; (v) developing the research protocol and logistics, obtaining ethics approvals and research permits, and training Country Office staff; and (vi) co-designing with communities in the demonstration projects.

By the start of Year 2 (June 2018), RISE had multidisciplinary teams and research platforms in place, communities enrolled, and was starting baseline data collection in Makassar. By year end, the 12 Indonesian main sites were randomised into intervention and control sites. As described below, delays in funding from the Asian Development Bank (ADB) slowed implementation in Fiji.

Year 3 (June 2019–May 2020) started off with completing community co-design in the intervention sites in Makassar, and baseline completion and then randomisation of the 12 sites in Fiji. The two demonstration sites were launched. Research teams in Fiji and Indonesia implemented field data collection, research management processes were institutionalised, and multidisciplinary coordination occurred organically and constructively.

Actual implementation varied from the original work plan. This was a result of: (i) operational delays and complexity in securing intervention funding; (ii) greater volume of work than anticipated to initiate the research (i.e. ethics, developing standard operating procedures etc.); and (iii) the demonstration projects were more work than originally envisaged, drawing resources away from setting up the core RCT.

## 4 Findings

Our findings (Table 1): point to several notable leadership and operational features and actions that proved useful to initiate and operationalise the program, as well as features and actions that were problematic. The findings are structured using the 11 principles of the Swiss Guide (Stöckli et al., 2018; Stöckli et al., 2018) for analysis and to align with literature and global best practice.

**Table 1:** Case study empirical findings analysed against the Swiss Guide (Stöckli et al., 2018).

Principles for effective north–south research	Key tensions	What worked	What didn't work
(1) Set the agenda together	• **The appropriate balance of decision-making authority and responsibilities between partners in the global north and south.** The program design phase (2016–2017) was largely based out of Melbourne, with limited engagement from Fiji and Indonesia researchers. This was not by choice, but shaped by various factors in the pre-award design phase: (i) the emergent, uncertain and time-sensitive nature of funding opportunities; (ii) our newness as a consortium and research group (including north–north relationships); (iii) the uncertainty of project locations until quite late; (iv) the complexity of the transdisciplinary research design; and (v) limited existing institutional partnerships in Fiji and Indonesia to build off. • **The randomised controlled trial (RCT) research design** necessitated pre-determined methods and parameters that limited opportunities for emergent research methods during implementation (discussed in detail below under principle 8).	• Restructuring the program budget, organisational structures and systems to expand the involvement and decision-making of researchers from Fiji and Indonesia. • Expanding the teams in Fiji and Indonesia, including new dedicated roles of Assessment Team Leader and Intervention Team Leader in each country. • Expanding the number and range of analyses undertaken in Country Office laboratories. • Bringing Fiji and Indonesia research leaders onto the core Leadership Team.	• Insufficient role of leaders from Fiji and Indonesia in the original research design decision-making. • Most implementation resources and responsibilities were originally allocated to global north universities, with Fiji and Indonesia partners responsible for community engagement and field sample/data collection and processing.
(2) Interact with stakeholders	• The program's complexity – stemming from its transdisciplinary and RCT features – proved difficult to reconcile with non-academic stakeholder expectations, motivations and timeframes. RISE involves multiple disciplines, datasets and research questions, over a long time period (initially five years, likely now to be closer to 10 years). The RCT need for a split between control settlements and intervention settlements, has proved challenging to communicate and secure buy-in, especially from local authorities and communities. • **Fiji and Indonesian government engagement and support was essential, yet it has been challenging to secure and maintain.** The range of government partners is broad and relevant functions are institutionally separated across Ministries and departments. We have experienced high levels of staff turnover in partner organisations, at political (i.e. Mayor), senior and technical levels. There have been variable levels of scepticism in Fijian and Indonesian authorities towards the program. These factors meant RISE leaders were constantly ‘re-pitching’ and socialising/re-enrolling government stakeholders to maintain momentum and garner support.	• **A unified implementation delivery ‘enterprise’ in each country** to foster and maintain effective non-academic stakeholder relationships. Distinct systems, processes, dedicated office space and the mandate to represent the full RISE consortium with external stakeholders. • **Country Office leadership and management team** (Country Leader, Country Manager, Assessment Team Leader and Intervention Team Leader) responsible and empowered to engage with all local stakeholders. • **Targeted investment in partnership managers/brokers at critical times** to advance the intervention, monitor the political economy of the context and draw on local networks and connections. • **Unified country-level assessment and intervention** and consolidated research assistants for all health research. • **Messaging for various audiences was co-developed** across the RISE consortium, drawing deeply on local insights and knowledge. • **Extensive face-to-face engagement** to develop mutual understanding and strengthen partnerships with local government partners through hands-on sustained engagement to build trust.	• **Insufficient resourcing for stakeholder management** given the scale and complexity of RISE, a program with 28 core partner institutions operating across multiple countries with a highly complex intervention. • **Underestimated the time required to enrol and secure support from government authorities**, for example for securing ethics, building and planning approvals for the intervention. • **High staff turnover in partner organisations** led to the need for re-pitching and re-enrolling stakeholders. • **Intervention funding modality** muddied the waters of who was responsible for enrolling local stakeholders and what could be said and promised (i.e. timeframes, funding scope and intervention design).
(3) Clarify responsibilities	• **The difficulty of leading and managing such a diverse consortium of partners across sectors and countries.** It was a brand new ‘coalition of the willing’ committed to an ambitious vision. Aligning divergent partner motivations, expectations, contributions and timeframes during implementation was challenging, as was synchronising the intervention and assessment work packages, which could not be de-coupled. Sequencing their associated investments from different funders proved difficult. • **Securing the intervention funding.** ADB had pledged to fund the intervention in both countries. However, as implementation progressed it became clear that this funding would not eventuate on a timeline and format that matched the RCT and expectations of other consortium members, first in Fiji in Year 1 and then in Indonesia in Year 2.	• **Secured alternative intervention funding for the control sites**, enabling RISE to continue. Executive and Leadership ‘held the uncertainty’ and leveraged the demand, goodwill and enthusiasm of local government and community stakeholders to articulate the need. • **Co-developed and socialised program-specific policies and guidance** applicable to all partners, and a code of conduct outlining inseparable responsibilities and ethical and moral values for all partners and individuals to follow. • Five-year institution-to-institution partnership agreements outlined roles, responsibilities and funding flows. • **Co-developed one common annual workplan**, along with holding **annual workshops** and regular interactive sessions to promote alignment and ensure coordination across multiple partners.	• Naïve about ADB's ability to partner in such a large consortium, for which we ultimately were unable to secure financing. • Responsibilities and accountabilities for northern researchers was not sufficiently articulated initially. • **Balance between individual researcher engagement vs. broader institutional partnerships.** This made it difficult to scale beyond peer-to-peer models of research delivery and impact.
(4) Account to beneficiaries	• Managing the divergence between what vulnerable informal settlement communities expect and want (rapid delivery of infrastructure) and the *raison d'etre* of RISE (research). RISE includes intensive continuous data collection, which requires high levels of community/resident participation and continued access. The RCT design requires setting study design variables at the outset, and controlling them during the study to compare intervention and control arms, which is difficult in real-world, dynamic settings such as informal settlements. • **How to responsibly implement complex transdisciplinary research in vulnerable informal settlement communities?** Was RISE something to be done *to* these communities, or was RISE something to be done *with* them? Given the flexible nature of the water sensitive intervention, how would RISE best communicate the intervention and be accountable to vulnerable informal settlement communities participating in the program?	• Co-developed clear protocols for initial community mobilisation (a structured nine-step process). • **Community Engagement Committees (CECs)**, one in each settlement, comprising local representatives to be the ongoing formal mechanism for program engagement, planning and delivery and community voice. • **Country Office teams included community development experts** as data collectors and fieldworkers, rather than only discipline-specific researchers, and built gender and ethnically diverse teams that reflected the study composition of study communities. • Clear communication material and protocols disseminated in English and the main local languages. • **Public randomisation ceremony** in each country. Attended by communities to feed back the baseline research findings and make explicit the random selection of intervention and control sites.	• **The exact scope of the ‘water sensitive cites’ (WSC) intervention was unknown at the outset**, which led to challenges in communicating and being accountable to communities for delivering an intervention which was so open ended and emergent. • Shifting timelines and responsibilities due to intervention funding. • The community partnership approach required more resources than originally budgeted for.
(5) Promote mutual learning	• **Balancing the achievement of ambitious delivery targets with sufficient time for deep mutual learning processes.** Meeting the funder-agreed timelines was a formidable leadership and management task. The workplan critical path had numerous interdependencies and uncertainties between the assessment and intervention. As a new program, significant time was also needed to formalise the partnerships and learn how to work together. • **RISE's transdisciplinarity placed significant burden on those involved for exchange and mutual learning.** Some research colleagues were frustrated by ‘slow/inefficient’ processes, including some early career researchers who were under pressure to ‘publish or perish’. Similarly, individuals working in non-academic partner organisations were often not willing to engage in deep dialogue and learning processes, which they perceived as academic, ‘ivory tower’ at the expense of delivery.	• **Joint trips, site visits and dedicated workshops** in Fiji and Indonesia nurtured multidisciplinary and cross-country exchange and learning. • **Annual workshops** and **annual report** provided structured opportunities for taking stock of what we had achieved, as well as reflecting on our experience to learn lessons. • **Fortnightly leadership meetings** provided a regular forum for information sharing and decision-making, testing new ideas, drawing from previous experience and indirect mutual learning. • **Country Office team debriefs** after each data collection campaign with structured documentation and feedback for improvement next time. • **Integrators group** to coordinate, learn and plan; weekly ‘stand up’ meeting; and a weekly internal email from Program Manager to all staff.	• **Some researchers** on RISE found the de-facto learning-by-doing approach difficult. • **Non-academic partners** were frustrated by the deep reflection, which was perceived to be at the expense of delivery and results. • Internal researcher frustrations were exacerbated by the **matrix organisational structure** and a lack of formalised knowledge flow systems in the early years. • Leadership and management paid **insufficient attention to culture building and norm setting** for effective mutual learning, and the University system does not have a fit-for-purpose pathway to accommodate the management support needed for mission-oriented transdisciplinary research.
(6) Enhance capacities	• **Reconciling the divergent views about what capacity development was and how it should be implemented.** An ongoing task was nurturing a common vision and understanding of capacity building as more than a one-way, north-to-south knowledge exchange endeavour. This was largely how the program and budget had been originally structured (see Section 4.1). This required re-visioning implementation plans in line with global best practice for multi-dimensional capacity building, and integrating ways of engaging with lived experience and non-Western knowledge systems in Fiji and Indonesia. • Securing sufficient time for dedicated capacity building, beyond core delivery activities, given the tight timeframe and so many competing priorities.	• **Institutional partnerships** with Fiji and Indonesia universities for sustainable knowledge production and use. • **Established Indonesia and Fiji research laboratories** and invested time in building the human capability of staff in each country, to localise research activities. • **Nurtured south–south cooperation** between teams in Fiji and Indonesia. • On the intervention side, we **developed and delivered training packages** on water sensitive design for local and central government staff and **used the demonstration projects as ‘living labs’** to build capability for the intervention.	• Capacity building activities were not funded. • **Shifting attitudes** away from capacity building as only a north-to-south endeavour. • **No explicit, structured approach/plan** to capacity building from the outset. • **No clear measurement framework** for tracking enhancement of capacities • **Insufficient formal mentoring and support** to junior scientists, including mentorship from global north leaders to global south leaders.
(7) Share data and networks	• Data sharing tensions emerged in three main areas regarding the integrity of the RCT, which aimed to provide proof of concept for a socio-technical solution for upgrading water-sanitation infrastructure in coastal informal settlements to reduce faecal environmental contamination and morbidity for those living in poor sanitary conditions globally: (i) sharing with research participants during the trial; (ii) data sharing across RISE teams to help design the intervention; and (iii) sharing and socialising improved water and sanitation behaviours (which could affect the trial outcome).	• **Developed research finding dissemination standard operating procedures,** to maintain the integrity of the RCT while also giving back to communities during implementation. • Reconciling tensions of internal data sharing to inform the design of the intervention.	• **Establishing a new transdisciplinary data system** that could operate across disciplines, institutions and geographies. • Managing highly sensitive human-subject data, particularly in an area of rapidly changing expectations and regulations regarding data management, security and privacy. This was compounded by some challenges in access to appropriate technology and infrastructure.
(8) Disseminate results	• **Divergent expectations on timeframe to deliver results.** Community and government expectations for early results, particularly given the perceived large quantity of data being collected, conflicted with the fact that the primary and secondary scientific outcomes will not be known until completion of the RCT.	• **The two demonstration projects** proved useful to disseminate results and lessons learned to non-academic partners without compromising the integrity of the main RCT, but the timeframes, complexities and costs of delivering these were markedly underestimated.	• Significant data and findings exist, despite the RCT not being complete, and which have not yet been fully harnessed to contribute to scholarship.
(9) Pool profit and merit	• **The size and complexity of the consortium, and change during implementation** complicated reaching agreement on – and keeping updated -–equitable profit and merit sharing. • **Practical research ecosystem differences between global north and south.** Collaborators in the global south often lack secure employment, are underpaid, and rely on top-ups (generally not features of global north researchers with relatively stable jobs and good salaries).	• **Unified communications and program identity.** We established a standalone identity, including a logo, style guide, website and public newsletter. This provided a common platform and identity that all personnel could align with regardless of their contracting institution. It proved particularly beneficial for teams in Fiji and Indonesia who were engaged with government and communities.	• **Agreeing on authorship** of academic outputs is a continual challenge that was difficult to manage at a program level due to divergent (disciplinary) cultures and norms.
(10) Apply results	• **Managing local demands for replication despite the RCT not being complete.** There was significant interest by Indonesian authorities, particularly the City of Makassar, to replicate/expand the water sensitive cities approach in other settlements, despite the RCT not being complete. Many researchers were uncomfortable with this as the scientific results on the efficacy of the approach were still unknown.	• **A series of three reports**, co-authored with the ADB and available in multiple languages, codified experience with the demonstration projects and supported local research to policy influence.	• **Insufficient funding for result application activities** to meet the demand from local partners for scaling up the approach, particularly in Indonesia.
(11) Secure outcomes	• **Funding is secured only for the defined program period**. There is no core funding to maintain the research ecosystems in Fiji and Indonesia beyond the program period.	• Investment in laboratories and research ecosystems in Fiji and Indonesia, including co-contributions from counterparts in these locations.	• **Insufficient diversification of funding** to sustain operations beyond life of program.

### 4.1 Principle 1: set the agenda together

A recurrent tension throughout RISE's first three years was finding an appropriate balance of decision-making control and responsibility between global north and south partners. The program design phase (2016–2017) was largely based in Melbourne, with limited engagement from Fiji and Indonesia counterparts. This was not by choice; rather it was shaped by various practical factors in the pre-award design phase: (i) the emergent, uncertain and time-sensitive nature of Wellcome and ADB funding opportunities; (ii) our newness as a consortium and research group (including north–north relationships); (iii) the uncertainty of project locations until quite late (i.e. which Indonesian city would be selected); (iv) the complexity of the transdisciplinary research design; and (v) limited existing institutional partnerships in Fiji and Indonesia to build off. Perhaps unsurprisingly, most implementation resources and responsibilities were allocated to global north universities (i.e. undertaking advanced environmental and human analyses) with the Fiji and Indonesia partners responsible for community engagement and field sample/data collection and processing.

The balance of decision-making control and responsibilities evolved depending on the demands of different phases and as capabilities and levels of trust grew. Within the first year, the Leadership Team restructured the program budget, organisational structures and systems to expand the involvement and decision-making of researchers from Fiji and Indonesia. An early shift was to have larger teams in Fiji and Indonesia, including a new dedicated role of Assessment Team Leader and Intervention Team Leader in each country. Similarly, as Country Office laboratory capability and capacity grew (RISE set-up new laboratories with equipment and hired and trained additional laboratory staff), the number and range of environmental and human microbiological analyses undertaken locally increased, and Fijian and Indonesian research leaders were brought onto the core Leadership Team.

Key enablers of these changes included leadership's openness to learn and adapt; regular/engagement for mutual learning (i.e. fortnightly meetings); a relatively flexible budget by Wellcome and Monash; the Program Manager's background and experience in development; strategic and sensitive advocacy from global south leaders and ‘boundary-spanning’ capability of mid-career ‘integrators’ advocating for greater involvement of Country Office teams; and spending significant joint time in Fiji and Indonesia to build trust, respect and friendships.

Overall, setting the agenda together was not ‘set and forget’ at the outset, recognising the need for adaptability. Global north leaders and managers were agile, collaborative and humble about their research capability in Fiji and Indonesia. The expanded roles of southern researchers and institutions as program implementation progressed increased mutual ownership and accountability and, despite the rigid boundaries of the pre-determined RCT (discussed below), strengthened transdisciplinary research design and methods.

### 4.2 Principle 2: interact with stakeholders

Principle 2, *Interact with stakeholders*, focuses on engagement with external, non-academic stakeholders to deliver the research and ensure up-take of the findings. As outlined in Fig. 2, key stakeholders included national governments in Fiji and Indonesia, local authorities (Province of South Sulawesi and city of Makassar in Indonesia, and four city councils in Suva), state-owned enterprises (Fiji Water Authority), research authorities (Indonesian Ministry of Research and Technology RISTEK), and communities/residents in the 24 informal settlements in the RCT (plus two demonstration projects).

While the RISE vision was compelling to external stakeholders, the program's complexity proved difficult to reconcile with diverse non-academic stakeholder expectations, who were short on time and motivated for quick ‘real-world’ impact. The RISE intervention is significantly more complex than conventional water, sanitation and hygiene (WASH) interventions (Wong et al., 2018). RISE involves land tenure regularisation, black- and grey-water management, community co-design, and flood protection. Similarly, the research design involves multiple disciplines, methods and research questions, and tight controls due to the RCT methodology. Furthermore, the RCT's need for control and intervention settlements, with a two-year gap between them receiving the intervention, proved challenging to communicate and secure buy-in, particularly from local authorities and communities. With the diversity of disciplines encompassed within RISE, developing a single unified voice to present to stakeholders and communities required time to develop.

Fiji and Indonesian government engagement and support was challenging to secure and maintain. The range of government partners was broad with relevant functions institutionally separated across Ministries and departments (i.e. health, environment, water and sanitation, urban planning and housing, and local government). We experienced high levels of government staff turnover, at political (i.e. Ministers and Mayors), senior and technical levels. Securing ethics approvals and material transfer agreements (MTAs) involved protracted processes, particularly in Indonesia, potentially stemming from a legacy of past poor experiences with researchers from the global north and/or lack of trust of our new partnership. Understanding Nagoya obligations was also complicated because each partner country had different frameworks and systems of governance depending on the samples collected and the type of analyses to be performed. RISE leaders spent considerable time and effort socialising/(re)enrolling and ‘re-pitching’ to government stakeholders to maintain momentum and garner support over the first three years of implementation.

Our findings point to the value of having one unified implementation delivery ‘enterprise’ in each country to foster and maintain effective non-academic stakeholder relationships. RISE Country Offices were set up as distinct new enterprises, with systems, processes, dedicated office space and the mandate to represent the full RISE consortium with external stakeholders using the RISE branding (see also below^2^). Each Country Office has a dedicated and explicit leadership and management team comprising Fiji/Indonesian nationals (Country Leader, Country Manager, Assessment Team Leader and Intervention Team Leader) responsible and empowered to engage with all local stakeholders. The core leadership team was supplemented by targeted investment in partnership managers/brokers at critical times to advance the intervention, and experts in the political economy, context and local networks. We unified activities under each Country Office leadership team, and consolidated research assistant responsibilities for all assessment-related work, thereby bringing coherence and efficiency to the program delivery mechanisms. The messaging for various audiences was co-developed across the RISE consortium, drawing deeply on local insights and knowledge. It was also helped by northern-based program staff and researchers spending significant time in Fiji and Indonesia (i.e. up to 4 months cumulative per year); this face-to-face engagement was crucial for mutual understanding.

The most significant stakeholder engagement challenge was the stewardship of the ADB intervention funding, which ultimately was not secured for the main sites. The reasons for this were complex and multifaceted, but essentially we did not explicate and adopt sufficient mitigation strategies for the significant (existential) risks to the program associated with a funding partner whose funding was pledged but not yet secured. We also had insufficient resourcing for stakeholder management given the scale and complexity of RISE, and we significantly underestimated the time required to enrol and secure support from government authorities to obtain ethics, building and planning approvals. Timeline expectations held by global north institutions proved unrealistic, which may have exacerbated the situation by inadvertently appearing as being ‘rushed’, thus fuelling sentiments about extractive, unequal north–south research and undermining trust.

### 4.3 Principle 3: clarify responsibilities

In terms of clarifying responsibilities, we find a key tension relates to the breadth of the RISE consortium, spanning a diverse group of partners across sectors and countries. Led by Monash University, RISE was designed with a carefully curated consortium of disparate partners from academia, industry, government, non-profit and international development actors (Fig. 1) based on each partner's comparative advantage, core competencies and pledged contributions (cash and in-kind). This ‘coalition of the willing’ committed to the ambitious vision of testing a new approach to water and sanitation servicing of informal settlements. However, aligning divergent partner motivations, expectations, contributions and timeframes during implementation was challenging. Many times during the start-up phase, we felt we were ‘building the plane while also flying it’. This metaphor captures the approach of undertaking long-term planning concurrent with experimentation and production, where details are worked out as implementation proceeds despite not having certainty around project boundaries and variables, and with a view to continual improvement while things are in progress. A notable example was the need to concurrently develop research protocols and enrol communities despite uncertainty regarding the intervention parameters and timeframes.

Ensuring ongoing accountability for pre-agreed responsibilities and contributions also created significant anxiety and tension, particularly challenges in accessing pledged intervention funding (to a value of US$10 m). No intervention funding meant no RCT research, and thus no program. Could RISE ethically and morally continue if intervention funding was in doubt? Ultimately, we secured alternative intervention funding from the New Zealand Government for Fiji and from the Australian Government for Indonesia for the 12 intervention sites, enabling RISE to continue. This was achieved by leveraging the demand, goodwill and enthusiasm of local government and community stakeholders through our (by that stage) strong Country Offices. But in the interim, the Executive and Leadership had to ‘hold the uncertainty’ to avoid reducing morale, losing other partners and putting the whole endeavour at risk.

A few key tactics and tools worked to align responsibilities and make partners accountable for their contributions. Co-developing one common annual workplan, along with an annual workshop and regular interactive sessions, were important for promoting alignment and ensuring coordination across multiple partners. A code of conduct outlined inseparable responsibilities and ethical and moral values for all partners and individuals to follow. Five-year institution-to-institution partnership agreements outlined roles, responsibilities, commitments and funding flows.

In retrospect, we were too optimistic about ADB's operational ability to partner in such a large consortium; it strayed too far away from their standard way of operating. While clarifying responsibilities at the outset was important, it was insufficient; timely fulfillment of pledged contributions is often much more complex and demands unique leadership and management capabilities.

Internally, across the global north, RISE's matrix structure was also challenging, with many university researchers acting on goodwill while balancing multiple research projects, teaching activities and academic service demands. There was insufficient clarity on certain day-to-day program responsibilities, and struggles balancing engagement with individual researchers in Fiji and Indonesia versus broader institutional conversations. On reflection, RISE started with the researcher-to-researcher model, which can be limited by field of research divides. For successful implementation from the outset, RISE needed broader and deeper institution-to-institution partnerships to operate at the enterprise scale in each country.

### 4.4 Principle 4: account to beneficiaries

Undertaking empirical research in informal settlements raises complex accountability questions. Informal settlements, also referred to as slums, are characterised by infrastructure deficits; poverty and social marginalisation; land tenure insecurity; and environmental hazards that climate change will likely exacerbate (Ezeh et al., 2017; French et al., 2020a). Theory, policy and practice over the past three decades have promoted ‘in-situ upgrading’ through regularising land tenure and improving infrastructure (French et al., 2020b). Prevailing community expectations are for foreign aid or government programs to pave streets, install drainage and build new water and sanitation infrastructure relatively quickly.

Given the divergence between what the communities expect and want (rapid delivery of improved infrastructure) and the *raison d'etre* of RISE (long-term innovation research), RISE's accountability to vulnerable informal settlement communities participating in the program has been complex from the outset. The research element requires intensive and continual data collection (i.e. health and wellbeing surveys, stool collection, water and mosquito sampling etc.), which requires high community/resident participation. The RCT design required setting study design variables at the outset and controlling them during the study to compare intervention and control arms, which is difficult in dynamic informal settlements. This required careful consideration and compromise on a consensus approach to community messaging to maintain engagement without compromising scientific integrity, with the depth of messaging depending on discipline-specific researcher expectations. Sometimes, it also required enrolled communities to forego significant investment from other programs for upgrading. These issues demanded highly collaborative, humble, agile and reflexive capabilities of RISE research leaders and managers, and Country Office leaders and staff.

Tactically, we drew heavily from international development methods and tools. We co-developed (with partners and communities) clear protocols for initial community mobilisation (a structured nine-step process). We formed Community Engagement Committees (CECs), one in each settlement, comprising representatives as the ongoing formal mechanism for engagement, planning and delivery. For Country Office staff, we hired community development experts as data collectors and fieldworkers rather than disciplinary-specific research staff. Our gender and inclusion action plan aimed for 50/50 male/female staff ratios in Fiji and Indonesia and built ethnically diverse teams to ensure teams reflected the study communities. We developed and disseminated clear communication material and protocols to all residents in English and the main local languages. Following baseline data collection, we held a public randomisation ceremony in each country attended by communities, to feed back the baseline research findings and make explicit the truly random selection of intervention and control sites.

The most significant community-level accountability challenge was that the exact scope of the ‘water-sensitive cites’ (WSC) intervention was unknown at the outset and needed to be tailored to each site's specific environmental and health stressors. Challenges arose in effectively communicating to communities that we would deliver an open-ended and emergent intervention. Community partnership approaches required more resources than originally intended, necessitating additional investment for community engagement.

### 4.5 Principle 5: promote mutual learning

The ambitious delivery targets had to be balanced against time for deep mutual learning processes, creating a formidable leadership and management task. Significant demands were placed on those involved for multidisciplinary and cross-sector exchange and mutual learning given divergent jargon, conceptual frameworks and epistemologies. Further exacerbating the problem, several early career researchers were under pressure from the ‘publish or perish’ system yet by necessity were spending the bulk of their time managing operational activities, making the transdisciplinary engagement seem frustratingly complex and slow. Similarly, individuals in non-academic partner organisations were not sufficiently willing to engage in deep ‘ivory tower’ dialogue and learning processes. We underestimated the challenges of facilitating effective mutual learning on this scale (daily, weekly, monthly, annually), while also balancing core ‘non-academic’ delivery demands. In hindsight, we underestimated the numerous interdependencies and uncertainties of the workplan's critical path, as well as the pressure to show early results that stemmed from running a flagship program with a notable public profile through media coverage and interest.

RISE, therefore, was characterised by a de-facto, emergent ‘learning-by-doing’ culture. Numerous joint trips, site visits and dedicated workshops in Fiji and Indonesia proved instrumental in nurturing multidisciplinary and cross-country exchange and learning. The annual workshops and annual report provided structured opportunities for taking stock of what we had achieved, as well as reflecting on our experience to learn lessons and improve. Fortnightly leadership meetings provided a regular forum for information sharing and decision-making, testing new ideas, drawing from previous experience and indirect mutual learning, to build the leadership team culture. Once established, Country Offices led structured reflection and learning, notably team debriefs after each data collection campaign with structured documentation and feedback for improvement next time.

However, not everyone embraced this ‘learning-by-doing’ approach, and a lack of established and formalised knowledge flow systems exacerbated certain frustrations. We overcame some communication and system flows by establishing an ‘integrators’ group to coordinate, learn and plan. This group was made up of students, early career researchers and mid-career researchers working on the program, as well as key members of the Country Offices performing day-to-day activities. Regular ‘integrator’ meetings helped create structure, but highlighted that we paid insufficient attention to culture building and norm setting for effective mutual learning.

### 4.6 Principle 6: enhance capacities

Initially, we did not have an explicit, structured approach to capacity building. A principal tension was reconciling the divergent views about what capacity development was and how it should be implemented. We had to sensitively challenge the initial project design and budget, which was largely structured as one-way, north-to-south knowledge exchange. Instead, we had to re-vision implementation plans in line with global best practice for multi-dimensional capacity building, and build a culture that recognised everyone was on a learning journey. Similarly, we did not set out to measure the enhancement of capacities explicitly; this was a missed opportunity, and should have been a dedicated and funded element of the original proposal.

Our recognition of these deficiencies in planning led to us incorporating several strategies and tactics that proved beneficial. We established long-term institutional partnerships with Fiji and Indonesian universities; funded and built research laboratories in each country; and invested time and funding to build the capability of laboratory staff so they could undertake most of the environmental and medical analyses. We nurtured south–south cooperation between teams in Fiji and Indonesia to expand the scientific potential of regional networks. We also developed and delivered training packages on water sensitive design for local and central government staff and used the demonstration projects as ‘living labs’ to build local capability.

### 4.7 Principles 7–11

Principles #7 to #11 of the Swiss Guide reflect the later stages of the research cycle, so we have fewer case study findings from RISE Years 1–3 for them. Regarding Principles 7 and 8, Share data and networks and Disseminate results, we find three significant tensions which reflect diverse perspectives on how to maintain the scientific integrity of the RCT.

First, there were divergent views on approaches to sharing data and early findings with research participants during the RCT. Leaders and managers had to navigate a broad continuum of (what became quite entrenched) views. For many researchers, sharing the scientific data during the trial could affect the trial integrity by changing community behaviours in a way that could affect trial outcomes. Some researchers, with a pragmatic approach, were willing to share only enough data/results to retain access and keep communities enrolled. Others were proponents of citizen science approaches, setting up direct conflict with those at the other end of the continuum that saw the importance of not sharing data and results during the trial period.

Second, there were also tensions regarding sharing baseline data across the investigator team, as this could potentially influence the intervention design and thus undermine the RCT. These differences, deeply rooted in disciplinary norms and epistemologies, were a continued tension that required careful negotiation to find common ground. Additionally, there were different viewpoints on the information that could be shared without compromising ethical obligations related to data confidentiality and privacy. Practical realities also compounded this challenge, including building a data system that could manage and integrate interdisciplinary data across institutions and geographies while ensuring the security of highly-sensitive identifiable human data on participants.

Third, the demonstration projects led to significant interest by Indonesian authorities, particularly the City of Makassar, to replicate/expand the water sensitive cities approach in other settlements, despite the RCT not being complete. Many researchers were concerned about early replication because the scientific results from the RCT on the efficacy of the approach were still unavailable. Ultimately, we found different parties simply had to ‘agree to disagree’ on some issues so we could move forward.

Related to Principle 9, *Pool profit and merit*, a key tension was the large size and complexity of the RISE consortium, and member changes during implementation. These factors complicated the process of reaching agreement on – and keeping updated – equitable profit and merit sharing. The unified communications and program identity helped navigate this challenge. Establishing a standalone identity, including a logo, website, newsletter and community engagement tools, provided a common platform and identity that all personnel could align with regardless of their contracting institution, and that ‘lived’ beyond any one partner. It proved particularly beneficial for teams in Fiji and Indonesia who were engaged with government and communities; the branding gave professionalism and was a shorthand for the overall endeavour.

Consistent with existing scholarship, authorship of academic outputs remains a major challenge. Authorship is not easy and our experience is that leaders and managers often cannot completely reconcile divergent views, cultures and norms across disciplines and geographies. We developed an Authorship Policy that sets minimum standards and encourages co-authorship with those who have made a significant intellectual and/or material contribution to the conceptualisation, implementation, analysis and writing activities. But the nuances of who should be included, how to account for student needs and how to appropriately recognise non-traditional academic contributions often require individual negotiation.

## 5 Discussion

### 5.1 Leading and managing north-south research and innovation

Our case study highlights the complexities and tensions facing research leaders and managers implementing large, multi-partner, north–south transdisciplinary programs researching a ‘wicked’ sustainable development problem. Consistent with recent scholarship (Wardani et al., 2022; Hoffmann et al., 2022a), difficulties arose from structural constraints set in the pre-award phase, including complex funding structures and grant requirements, the research design, and the matrix organisational set-up of delivery activity across multiple institutions (Schmidt and Pröpper, 2017). During implementation, these proved difficult to reconcile with ambitions for a responsible north–south research praxis, as outlined in the Swiss Guide. This factor reaffirmed the importance of having sufficient pre-award time and commitment for meaningful co-design to develop initiatives (Newman et al., 2019), building on pre-established partnerships and networks where possible – what O'Donovan et al. (2022) refer to as ‘*backstage capability’* (Adler, 2009; Pohl, 2005).

As similar research found (Saric et al., 2019; Cundill et al., 2018; Hoffmann et al., 2022a), ‘boundary objects’ – commonly shared and understood items spanning across geographic, disciplinary and stakeholder boundaries, such as the annual workplan – proved effective leadership and management tools, especially when managed flexibly to adapt to different needs, users and changing contextual conditions. Active, agile management using these tools can help to overcome the collaboration and accountability difficulties posed by matrix structures and multiple partners. Hollaender et al. (2008, p.387) noted “… active management is very important for the success of transdisciplinary teams. A laissez-faire type of leadership, which hopes that the different parts of the work of transdisciplinary teams will grow together organically has not proven successful.” Similarly, drawing from four thematic synthesis processes of the Swiss National Research Programme (NRP 61) on Sustainable Water Management Hoffmann et al. (2017, p.678) find “transdisciplinary integration requires professional competences, management skills and enough time”. The authors suggest “fostering communities of practice (CoP) to link committed leaders and enable mutual learning processes beyond the boundaries of individual synthesis projects or research programs.”

The findings shine light on emerging scholarship on the relational human-to-human aspects and individual capabilities needed for creative implementation (Cundill et al., 2018; Lyall et al., 2015; Boone et al., 2020). Echoing Cundill et al. (2018), our findings point to the value of forming friendships and strong bonds across cultures as part of reflexive, mutual learning processes. Principle 3 in Hoffmann et al.'s (2022a, p.965) 21 principles for leading, learning and synthesising inter- and transdisciplinary research is “forge social bonds', to ‘facilitate socioemotional relations among highly diverse group members and craft a productive group culture and identity.’ Our findings regarding the value of face-to-face engagement in ‘the field’ echo Bergmann et al.'s (2021, p.557) analysis of real-world-lab programs that offer opportunities “… to look at problems in physical space and provide occasions for informal exchange.” This can often help buffer the bad times and incentivise action more effectively than formal contracts and management systems.

This finding reflects calls for more humanistic, empathetic research leadership and management given the high social and personal costs to researchers in transdisciplinary projects and tendency for burn-out (Sellberg et al., 2021; Felt et al., 2013). Hoffmann et al. (2022a, p.965) find that “a small number of leaders, sharing or distributing leadership across their groups' members, may be more effective than a single individual in leading for creative synthesis.” The findings also show that experience matters (having undertaken similar research before). Experienced leaders and managers can draw on their embodied knowledge to be sensitive facilitators nurturing consensus (Clark and Stankey, 2006; Defila and di Giulio, 2017), and to operate in an environment of high uncertainty and unpredictability (Roux et al., 2010; Hoffmann et al., 2017). Mentoring and mutual learning to be ‘t-shaped’ researchers, who have both disciplinary depth and transdisciplinary breadth’, cannot be taken for granted (Brown et al., 2019; Hoffmann et al., 2022a).

Our findings extend scholarship by challenging the prevailing conceptual divide between leader and manager roles for complex north–south research. Consistent with recent research (Hoffmann et al., 2017, Hoffmann et al., 2022a; Larkan et al., 2016), we show the complexity of such research requires incredibly close alignment of the vision, strategic direction and research design with the day-to-day management decision-making and implementation. It is difficult to separate the vision from the execution of entrepreneurial, emergent research collaborations (Hansson and Mønsted, 2008; O'Donovan et al., 2022). In practice, research leaders and managers are often the same people due to limited resources, institutional accountability systems and academic culture and precedents, and as Hoffmann et al. (2022a) find, assuming the role of an integrative leader is challenging.

Recent scholarship points to the vital role of boundary-spanning ‘integrators’ – “experts who specialise in leading, administering, managing, monitoring, assessing, accompanying and/or advising others on integration within ITD [inter- or transdisciplinary] projects or programs” (Hoffmann et al., 2022b, p.2). Integrators translate academic vision to execution and nurture relational capabilities and spaces for cross-cultural and multidisciplinary learning, thus offering peer-to-peer mentoring across north-south researchers.

The findings show how ‘pracademics’ (Powell et al., 2018) and ‘integrators’, across the global north and south, span across sectoral, organisational and disciplinary boundaries to forge consensus and nurture a culture of collaboration across highly asymmetrical power dynamics. Our findings support claims that these roles make scholarly contributions beyond conventional research administration tasks (Hoffmann et al., 2022b), challenging the conventional model of managers and administrators doing the ‘back of house’ tasks, subordinate to the scholarly lead researcher(s) generating ideas and publishing the research (Nature, 2022), particularly in global south contexts to minimise the risks of a ‘helicopter research’ approach (Haelewaters et al., 2021). As König et al. (2013) noted, these professional roles have become more prevalent as north–south research programs have become larger in scale and complexity and are worthy of deeper attention and study.

Our findings reaffirm the critical importance of having leaders and managers from the global south and embedding a research program in the local southern context under study. Having P-CIs and CIs from the global south, with decision-making authority, rather than all CIs being from the global north, improves the research questions and design, and minimises intercultural misunderstandings and unexpected dynamics and diverging interests during implementation (Schmidt et al., 2018; Pohl, 2005; Schmidt and Pröpper, 2017; Wardani et al., 2022). Caution, however, needs to be given regarding tokenistic approaches, along with managing the ‘hidden burden’ on global south researchers (Luetkemeier et al., 2021). Having an appropriate balance of authority and responsibility between north and south is an ongoing process, not a ‘set and forget’ at the outset; it changes as trust, capability and capacity evolve during implementation. Importantly, capacity building of southern researchers and research ecosystems is an important responsibility for research leaders and managers, and should include research leadership and management capabilities not just scientific (Diez Roux et al., 2018; Felt et al., 2013; Carbonnier and Kontinen, 2015). Edejer (1999, p.1) noted “… scientific advances are not the only yardstick to measure the success of north–south research collaboration”; investment in local research capacity building is an important indicator of success.

As others have found (Zingerli, 2010; Hollaender et al., 2008; Hoffmann et al., 2022a), high levels of *trans*disciplinarity place unique demands on leaders and managers beyond the conventional (individual) research capabilities, and beyond standard organisational delivery systems and structures for mono- and inter-disciplinary research. As Hollaender et al. (2008, p.386) notes, transdisciplinary research's inherent heterogeneity makes it powerful, yet difficult to manage: this is the transdisciplinarity paradox. These issues are compounded when programs are multi-country, and involve a highly complex intervention, aspiring to achieve ‘real-world’ impact. Our findings provide pioneering evidence on the numerous challenges that arise for complex RCTs, explicating the difficulties of reconciling this research methodology with ambitions for responsible north–south research praxis (Stöckli et al., 2018; Reidpath et al., 2022; de Souza and Eyal, 2019).

Our findings also support scholarship on the ‘capability gap’ for research leaders and managers for this kind of research (O'Donovan et al., 2022). Significant additional capabilities are needed beyond those required to operate in one's own context (be it north or south), sector and discipline. The nuances of what these additional capabilities are, and how they manifest day to day, are largely silent in scholarship and guidance (i.e. the Swiss Guide). While not north–south, O'Donovan et al.'s (2022) framework for evaluating researcher capability improvements in transdisciplinary research projects reflects the multi-level nature: individual capabilities are important but in themselves insufficient; collective capabilities and contextual influences are also important in shaping implementation. Most literature and guidance implicitly posit these roles like robots, with unlimited time and resources implementing projects, rather than humans as social beings who must make difficult trade-offs, balance competing priorities, and navigate the shifting and opaque political economy of ‘real-world’, cross-cultural implementation. Our findings ‘bring to life’ the joined-up role of leader–manager and the tensions such people face in advancing complex transdisciplinary north–south research.

Our findings also highlight the role of research methodologies in setting the opportunities and constraints for transformational change. Transdisciplinary research that combines and integrates multiple methods, in collaboration with research participants, adds a layer of complexity that cannot be underestimated. Rigid methods such as randomised controlled trials, as this case shows, pose challenges of data sharing, engaging participants as partners (rather than ‘subjects’), and allowing flexibility to adapt to accommodate changes during implementation, which constrain potential impact for study communities during implementation. While critical examination of the implementation challenges and generalisability of RCTs (Reidpath et al., 2022) aligns with efforts to develop new ‘pragmatic trial’ methodologies (Zuidgeest et al., 2017), there remains a need for greater examination of methodological approaches, trade-offs, and best practices that can support aspirations for transformation change for the global south.

### 5.2 A framework for operationalising north-south transdisciplinary research

Table 2 presents a framework for leading and managing north–south transdisciplinary research. It outlines the individual skills, capabilities and capacities for leaders and managers along with organisational capabilities and enabling supports. The framework has five domains: (1) collaborative leadership; (2) agile management; (3) flexible consortia; (4) researcher positionality; and (5) co-design and participation. The Framework was generated through an inductive approach. Drawing from the specific findings on effective tactics and tools, and lessons learned compiled during the collaborative ethnography workshops, these were sorted and grouped in patterns, leading to a set of features and principles, and the five domains. The draft framework was interrogated with the author group to arrive at the final domains, each with features and principles, and tactics and tools for leaders and managers. The Framework is not intended to be exhaustive and we hope it can be enriched and expanded on by others in addition to ourselves as the RISE program advances.

**Domain 1: Collaborative leadership** refers to a more explicit kind of leadership that prioritises the role of the leader as a boundary spanner and facilitator. Leaders bring together people and organisations from diverse disciplines and boundaries to achieve technological and social innovation. Collaborative leadership also shares and decentralises control and authority beyond formal leaders, to promote effective collaboration and high team performance (Archer and Cameron, 2013; Hollaender et al., 2008; Hoffmann et al., 2022a). Transboundary north–south research endeavours to prioritise the co-development of the program vision, research design and problem solving with all partners (as outlined in Principles 1 and 2 of the KFPE Guide). It nurtures a learning-by-doing approach and flexible team structures, and enables the endogenous growth of leaders, especially early and mid-career researchers who are learning transdisciplinary skills and practices (Felt et al., 2013). Leadership capability can be nurtured through mentoring opportunities between established and early career researchers, as well as between collaborators in the north and south to develop skills and transfer knowledge in parallel with activity delivery.
**Domain 2: Agile management** breaks an overall work program into smaller discrete packages. These packages are often implemented in parallel through ‘sprints’, to innovate, experiment, test and refine, and feed lessons back to adapt to changing context needs or priorities (Sutherland, 2015). The agile approach empowers managers with a high degree of autonomy and responsibility and firmly embeds them in a nimble delivery ecosystem (Stark, 2014; Hoffmann et al., 2022a). They are facilitators rather than directors, keeping teams focused on the common overall goal and proactively coordinating the constituent workflows. Agile management requires effective and efficient communication systems (e.g. regular ‘stand up’ meetings to report on sprints) and significant investment in cross-cultural teamwork and exchange to build trust, cope with uncertainty, enhance capacity and promote mutual learning (Principles 6, 7 and 8 of the KFPE Guide).
**Domain 3:** Effective leaders and managers build and steward a **flexible consortium** of partners. They build trusted relationships with partners to form and maintain a ‘coalition of the willing’ committed to the overall goal (Stilgoe et al., 2013). They draw out partners' collective intelligence, harnessing their strengths, while paying explicit attention to the asymmetries of power and capability that exist between north and south consortium members (principles 3 and 4 of the KFPE Guide). Transdisciplinary programs can be most effective when they are anchored with a cross-faculty institute or centre that has the mandate and autonomy to lead transdisciplinary research, rather than within one particular school or faculty (Arnold et al., 2021; Brown et al., 2019). Similarly, co-locating intervention and control teams is uncommon, but facilitates knowledge exchange and rapid response to problems, as also found in a large WASH-Benefits trial in Bangladesh and Kenya (Unicomb et al., 2018).
**Domain 4: Researcher positionality** requires undertaking critical self-reflection on social identity and privilege to break down the traditional model of north–south research; i.e. northern ‘experts’ studying southern ‘subjects’ (Mellor, 2015; Lawson, 2007; Connell, 2007, Connell, 2014; Turner et al., 2024). Reflexivity is also required at the institutional level (Stilgoe et al., 2013; Wynne, 1993). And it includes people with lived experience, including in leadership and management positions, and foregrounds an intersectional lens to illuminate structural conditions that create and perpetuate inequalities in the research ecosystem (Wardani et al., 2022). Leaders and managers should recruit carefully and only engage people willing to commit to responsible research practice, and who are open to learning and improving international development research as part of a community of practice. Positionality also refers to the need to ‘think and work politically’, particularly in the global south where governance structures are less formalised and exhibit a greater number and heterogeneity in informal actors (i.e. traditional land owners, informal government representatives at community level) who can affect research design, implementation and uptake (Schmidt and Pröpper, 2017).
**Domain 5: Co-design and participation** requires leaders and managers to be comfortable with decentralised decision-making and resourcing, and involving global south collaborators in all stages – as partners, not as service providers or data collection hubs (Wardani et al., 2022). Uplifting the capability of global south universities and researchers must be an explicit aim, and financed appropriately (Cooke and Kothari, 2001). Meaningful participation requires long-term flexible partnerships founded on collective ambitions and a commitment to mutual learning, yet this is challenged by short-term project cycles and traditional research planning approaches (Schmidt and Pröpper, 2017).

**Table 2:** A framework for leading and managing large north–south transdisciplinary research.

Five domains	Features and principles	Tactics and tools for leaders and managers
1. Collaborative leadership	• Co-development of vision and practices with all staff/members, especially those in the global south • Collaborative problem solving, drawing on diverse experiences • A ‘learning-by-doing’ leadership approach, seeing the whole • Flexible team structures to foster innovation, experimentation and adapt to change • Diversity of team members in age, experience, gender, background, etc. • Everyone has responsibility for balancing strategic and operational matters	• Co-create the overall vision with all members through iterative workshops, joint field visits, discussion papers. • Implement dedicated capacity and capability workshops to develop capacity for collaborative leadership. • Establish and nurture formal and informal communications between formal and informal leaders. • Include leadership KPIs in annual performance plans to foster a collaborative leadership culture. • Co-develop and implement an internal gender and diversity action plan to mainstream inclusion across all activities and outcomes. • Pair senior scientists and staff with early and mid-career researchers for on-the-job mentoring and training. • Create and sustain a common research data team and system to track progress. • Decentralise financial resources and budgets to team leaders and groups. • Ensure language accessibility (not all in English). • Celebrate and showcase best practices and team members' roles.
2. Agile management	• Overall program divided into packages with sufficient autonomy (leaders and resources) • Packages implemented in parallel rather than in succession • Explicit ‘sprints’ to ideate, innovate, test and refine • Explicate continuous feedback loops between packages, locations and teams to adapt and incorporate lessons • Leaders and managers as ‘facilitators’ rather than directors, keeping teams focused on common goal • Responsive and proactive management of external partnerships	• Empower local teams to lead implementation (i.e. Country Offices), giving autonomy and resources to make adjustments in response to local context changes. • Delineate and be explicit about the two main role types: dedicated delivery roles with clear performance metrics, complementing in-kind research and advisory roles. • Ensure clear ToRs/position descriptions for all staff, especially package leads, and ensure sufficient resourcing for delivery. • Set up efficient communication systems and procedures within and across sub-teams. • Send weekly internal emails from leader/manager to all staff to disseminate key updates, decisions and progress. • Co-develop with teams, and use on a day-to-day basis, a common planning tool to show progress and align work packages (i.e. common workplan). • Facilitate weekly ‘stand up’ meetings to report on ‘sprints’ and adjust as required. • Invest in cross-cultural teamwork and exchange to build trust and cope with uncertainty and change. • Invest in frequent ‘all staff’ workshops to draw out lessons learned, provide feedback across packages and adjust implementation plans. • Ensure the research design is flexible to accommodate change. • Ensure financial/budget flexibility to accommodate changes, and communicate with funders the value of an agile approach. • Allow sufficient time for research ethics applications; use ethics applications and reporting as a tool for building internal consensus and understanding on the overall goal.
3. Flexible consortia	• Leaders and managers ‘crowd in’ partners to form and maintain a coalition of the willing committed to the overall goal • Leaders and managers draw out collective intelligence of partners, harnessing their key strengths across time and space • Not all members have the same role; transparent hierarchy of institutional partnership levels: lead, core and advisory/support • Adapt consortium partnership based on performance and as program needs change • Recognise the asymmetries in power and capability of north and south consortium members	• Anchor the program with a cross-faculty, institute or centre with the mandate, expertise and autonomy to lead transdisciplinary research. • Sign long-term institutional partnership agreements that have a degree of flexibility and are underpinned by trust; avoid a service-provider model with the global south. • Sign MoUs with non-academic partners for institutional anchoring and commitment (i.e. with governments, industry). • Conduct periodic bilateral ‘health checks’ with partners, taking corrective action when necessary. • Build the communications capacity of researchers so they can speak to different audiences beyond academia (government, communities etc.). • Foreground the role of global south institutions in the consortium in internal and external communications. • Implement systems to overcome high staff turnover in partner government agencies. • Create a shared program identity and external image that all partners can identify with (i.e. logo, website). • Create long-term collective value for the consortium, e.g. through ISO9001 quality assurance accreditation.
4. Researcher positionality/reflexivity	• Self-reflection on social identity, privilege and background to understand power relations and how to address these in a responsible manner • Northern researchers not seeing themselves as ‘the expert’ and participants in the global south as ‘the subjects’ • Sharing power and resources in the project cycle and knowledge creation chain, including non-western epistemologies and knowledge practices • ‘Thinking and working politically’ (TWP), grounded in strong political analysis and response to local context • Proactively nurture a constructive internal culture of reflexivity at various program stages	• Implement a Code of Conduct that all researchers sign up to establishing base expectations and principles of engagement. • Encourage all researchers to develop a positionality statement that outlines the lenses they bring to the endeavour. • Facilitate multidisciplinary and cross-cultural workshops to reflect and share positionality statements to build trust and enhance teamwork. • Include people with lived experience of the research area under study, including in positions of authority. • Foreground an intersectional lens to explain and account for how multiple social identities can coincide to create systems of inequality. • Select staff/researchers carefully. Engage only people who can commit to responsible research practice and are passionate about the overall goal. • Avoid inheriting staff, and ensure all staff have a clear ToR with KPIs that include responsible north–south research praxis and exchange. • Facilitate capacity development opportunities for global north researchers on international development theory and practice to provide a foundation for effective north–south engagement. • Articulate mentoring targets in annual performance plans to promote on-the-job capability uplift. • Build a community of practice showing good examples and practices of north–south research that confront the asymmetries of power and visibility. • Undertake in-depth analysis on the context under study to inform decision-making and course correction.
5. Co-design and participation	• Decentralise power and empower program staff/offices in the global south to be involved in all stages, as partners not as service providers • Include broad range of stakeholders beyond academia in the research cycle to align the research with its local context • Build in sustainability and longevity of outputs and outcomes for lasting impact • Make explicit the capability uplift of global south universities and researchers as an explicit aim of the activity, not an optional add-on	• Allocate significant amount of the program budget to Country Office teams and operations in the global south. • Build and nurture strong, diverse, qualified teams in the global south with a range of skills beyond research, and sufficient autonomy to drive implementation. • Foster long-term, flexible partnerships founded on collective long-term ambitions rather than short-term, project-based service-provider arrangements. • Have strong, full-time, well-paid leaders and managers from the study context, leading Country Offices and with comparable decision-making power as leaders from the global north. • Anchor with global south institutions whose decision-making authority is given equal weighting to global north institutions. • Hold annual workshops with partners to co-create work plans and exchange lessons. • Implement flexible staffing arrangements for knowledge transfer (i.e. secondment across institutions, staff embedded in partner organisations, extended study tours and trips etc.). • Produce collateral/training/briefing materials in accessible languages/formats to reduce time for retraining and facilitate meaningful engagement. • Establish and maintain dispute resolution and grievance mechanisms that work across cultures. • Develop realistic work plans that explicitly schedule in learning and capacity development activities for all researchers.

Co-design can be facilitated through annual workshops, co-created work plans and flexible staffing arrangements for knowledge transfer (i.e. secondment across institutions and embedding staff in partner organisations), and by scheduling sufficient time for learning and capacity development for all. Importantly, co-design also involves communities as active participants in research, at all stages; they are not subjects to be studied (Keikelame and Swartz, 2019).

Overall, the five domains are complementary to the 11 principles of KFPE's *Guide for Transboundary Research Partnerships* (Stöckli et al., 2018). While the Guide is structured chronologically, with an implicitly project lifecycle frame (i.e. set the agenda together (Principle 1) through to secure outcomes (Principle 11), these five domains are cross-cutting throughout the lifecycle of a research project. For example, collaborative leadership (Domain 1) and researcher positionality and reflexivity (Domain 4) are important at all project stages. Thus, the utility of this framework is its focus on the underlying leadership and management ethos and capabilities needed in addition to the project stage-specific needs as outlined in the KFPE Guide.

### 5.3 Implications

The implications from these findings point to the need for requiring all stakeholders supporting/implementing research and innovation aligned with the SDGs to accept and plan for longer program timeframes, a higher risk appetite and more agile delivery approaches with shared decision-making and governance mechanisms. Universities, home to much of the world's academic SDG research, need more flexible organisational structures and incentive systems that encourage collaboration across disciplines, countries and partners (Scholz, 2020).

Academic performance metrics and targets must evolve to balance scientific outputs against recognising the additional time and energy required for north–south engagement. As our findings show, universities would do well to break down the conceptual (and often contractual) divide between (academic) leaders and (professional) managers, most notably the ‘pracademic’ model (Powell et al., 2018; Hoffmann et al., 2022b). We recognise these reforms are not insignificant compared with ‘business-as-usual’ global north research ecosystems. Low-hanging fruit would be developing new professional development trainings to build capabilities as outlined in the framework and other guides.

The findings have implications for global research funders: adequately resourcing pracademic and integrator roles in programs, ensuring sufficient time for meaningful co-design, and using the project budget as a tool to ensure joint north–south decision-making and accountability are levers that may support responsible north–south research. Additionally, funders should recognise the value of supporting longer-term projects that deliver transformational change, and the inherent time (and costs) required for set-up, partnership-building, research operationalisation and capacity development, and provide sufficient resources to maximise sustainability potential.

Furthermore, our Framework has implications for efforts towards more responsible north-south research practices (Erondu et al., 2021; Haelewaters et al., 2021; de Souza and Eyal, 2019; Schroeder et al., 2020). Global north researchers should refrain from a ‘charity mindset’ and position partnerships within a rights-based approach to ensure southern researchers' equal role in co-owning research agendas, implementation and benefits (Nature, 2022). This requires shifts in the view of southern researchers' roles framed largely in terms of ‘fieldwork’, data collection, and local gatekeeping, and necessitates critical reflection on ‘fly-in, fly-out’ models that can reinforce power asymmetries and limit learning and effective synthesis. For researchers in the global south, the framework advocates for clearly outlining collaboration values and expectations at the outset and communicating how motivation is also linked to meaningful participation, autonomy and decision-making power. Co-develop frameworks for equitable research publishing, unapologetically advocate for a multilingual research environment, and make explicit and secure sufficient resourcing for local capability building learning.

Our study has three notable limitations. First, the collaborative autoethnography method excludes broader codification and analysis of experience from others involved in RISE. Second, the collaborative autoethnography method was not immune from the power imbalances and inequalities it sought to explore. Third, the findings relate to only the first three years of RISE and so should be read in context of the implementation establishment phase.

## 6 Conclusion

The number, scale and ambition of transdisciplinary SDG-focused research programs between global north universities and the global south is increasing. Yet, there is very little theoretical or empirical scholarship on how to responsibly lead and manage such endeavours. This study contributes a framework with five domains to support the design and implementation of future programs. We hope the framework principles and tactics can be added to over time and enriched with additional experience and insights.

The findings reaffirm the importance of research leaders and managers carefully and explicitly operationalising north–south research in a just manner, critically reflecting on power asymmetries between partners and locations, leveraging the potential for transdisciplinary consortia to build research capabilities in the global south, and creating a culture of reflexivity on the historical and social positionality in which research is designed, funded, implemented and evaluated. The findings foreground the role of ‘integrators’ and ‘pracademics’, roles that have received little attention to date but are essential for effective delivery and societal impact beyond scientific advances from research projects. Codifying this experience and knowledge and using it in future efforts can help minimise the pitfalls of a neo-colonial approach to research and innovation, drive lasting change, and build the research and scientific innovation capability needed to meet the SDGs by 2030.

## CRediT authorship contribution statement

**Matthew A. French:** Writing – review & editing, Writing – original draft, Visualization, Methodology, Investigation, Formal analysis, Conceptualization. **S. Fiona Barker:** Writing – review & editing, Investigation, Conceptualization. **Amelia Turagabeci:** Writing – review & editing, Investigation. **Ancha Ansariadi:** Writing – review & editing, Investigation. **Autiko Tela:** Writing – review & editing, Conceptualization. **Diego Ramirez-Lovering:** Writing – review & editing, Funding acquisition. **Fitriyanty Awaluddin:** Writing – review & editing, Investigation, Conceptualization. **Ihsan Latief:** Writing – review & editing, Investigation. **Isoa Vakarewa:** Writing – review & editing, Investigation, Conceptualization. **Ruzka R. Taruc:** Writing – review & editing, Investigation, Conceptualization. **Tony Wong:** Writing – review & editing, Funding acquisition. **Brett Davis:** Writing – review & editing. **Rebekah Brown:** Writing – review & editing, Investigation, Funding acquistion, Conceptualization. **Karin Leder:** Writing – review & editing, Methodology, Investigation, Funding acquisition, Conceptualization.

## Declaration of competing interest

The authors declare the following financial interests/personal relationships which may be considered as potential competing interests: Rebekah Brown reports financial support was provided by Wellcome Trust. Karin Leder reports financial support was provided by National Health and Medical Research Council. If there are other authors, they declare that they have no known competing financial interests or personal relationships that could have appeared to influence the work reported in this paper.

## Data Availability

No data was used for the research described in the article.

## Appendix A Supplementary data

Supplementary material Image 1

## Footnotes

1 The 11 principles include: (1) Set the agenda together; (2) Interact with stakeholders; (3) Clarify responsibilities; (4) Account to beneficiaries; (5) Promote mutual learning; (6) Enhance capacities; (7) Share data and networks; (8) Disseminate results; (9) Pool profit and merit; (10) Apply results; and (11) Secure outcomes.

2 This contrasts with many prevailing north–south research models, such as peer-to-peer-led researcher engagements, common in smaller-scale mono- and inter-disciplinary research. Therefore, it was RISE engaging with government rather than only Universitas Hasanuddin or Fiji National University (as the local representatives of RISE).
