# Supplementary material for: Responsible north–south research and innovation: A framework for transdisciplinary research leadership and management
Source: Res Policy. 2024 Sep;53(7):105048. doi: 10.1016/j.respol.2024.105048 (PMC11245643; doi:10.1016/j.respol.2024.105048)
Supplement: Supplementary file 1 — Supplementary material [file mmc1.docx]

## Supplementary Material 1: Use of the terms global north / global south

‘Global south’ is a term used to denote low- and middle-income (LMICs) countries mostly located geographically south of the equator in Africa, Asia, Latin America and Oceania, distinguished from the industrialised and higher-income countries of North America and Western Europe (the ‘global north’). The terms ‘global south’ and ‘global north’ have tensions worthy of highlighting, including: (i) they are descriptively incorrect (i.e. New Zealand and Australia are located in the south); (ii) they can foster a misleading binary that homogenises and masks significant social and economic diversity in countries’ levels of development and characteristics (Dados and Connell, 2012). They can reinforce the primacy of Western epistemological theories, eurocentric norms, parochialisms and academic dependency (Lawson, 2007; Connell, 2007; Connell, 2014; Cooke and Kothari, 2001). And they do not account for the recent blurring of north–south geopolitical boundaries notably the rise of BRICS countries (Brazil, Russia, India, China and South Africa) (Horner, 2020).

Despite these acknowledged limitations and tensions, we – like many other scholars – use the terms ‘global north’ and ‘global south’ for several reasons. We use it in this paper as an analytical category, one that foregrounds the critical theory elements that encourage critical reflection of our experience. It can expose asymmetrical power imbalances, and “… references an entire history of colonialism, neo-imperialism, and differential economic and social change through which large inequalities in living standards, life expectancy and access to resources are maintained” (Dados and Connell, 2012, p.12). The framing thus encourages critical reflection on north–south research processes shaping power, resources and the motivations underpinning research and action, and challenges de-facto assumptions that knowledge and scientific research expertise primarily flows from north to south.

## Supplementary Material 2: Why undertake a case study on RISE?

Given its scale, complexity and heterogeneity, RISE is an opportune case to explore the leadership and management aspects of transdisciplinary, transboundary, global north–south research. Characteristics that make it worthy of empirical investigation include: the large number of institutions involved (n=28), the range of countries (n=5, including 2 in the global south), and the number of people working on RISE (n=170 with half full-time). Another four important characteristics make RISE a potentially powerful empirical case of leadership and management demands of such research.

First, RISE not only observes ongoing phenomena but involves the direct delivery of a tangible, intensive development intervention. The intervention design is complex and has many components (i.e. water, sanitation, flood management, drainage, accessways). The intervention is new in Fiji and Indonesia. Informal settlements present particular difficulties including land tenure issues, high densities, social conflict, and multiple and overlapping vulnerabilities of study participants. RISE also implemented a demonstration project in each country to test and exhibit the intervention and co-design process, separate to the main study sites, which took considerable effort. The ADB pledged intervention funding, but it could not be unlocked, and the civil works needed Fiji and Indonesia government approvals.

Second, the RISE research design is a randomised controlled trial (RCT), which places unique conditions on operationalising the research compared with other research methodologies and collaborative initiatives. Delivering the intervention to control and intervention sites must be phased, with a two-year monitoring gap between to assess impacts. Six settlements in each country will receive the intervention at the outset; the others will serve as controls and be offered intervention delivery after trial completion. This presented unique challenges around managing study participant, community, donor and government expectations and participation. Similarly, the positivist and experimental nature of the RCT design demands developing and adhering to strict protocols, which do not always match community expectations, timelines and motivations.

Third, RISE is highly transdisciplinary with a broad range of disciplines and fields of practice: architecture, urban studies, engineering (Objective 1); biology, ecology, environmental science, microbiology (Objective 2); and public health, medicine and economics (Objectives 3, 4 and 5). Compared with other north–south programs that may stay largely within the HASS and STEM spheres, this disciplinary diversity presents both deep epistemological divergences as well as everyday operational challenges around managing diverse approaches, norms, language and methods.

Fourth, RISE started from scratch, unlike many other north–south endeavours which grow from previous smaller projects and exchanges. Most RISE members had not worked together before, and many researchers from RISE’s global north universities had not worked in the global south before joining the program. While sub-teams brought their discipline-specific research tools and protocols from previous projects, there were no established program-wide operational guides, manuals and tools to promote transdisciplinary collaboration. The Fiji and Indonesia offices and laboratories were established, and country teams recruited, formed and trained from scratch.

These attributes created significant demands on all members, particularly RISE leaders and managers. As mentioned above, existing scholarship is nascent on *how* to do effective north–south research overall, and even more nascent on north–south research with this level of complexity.

**Supplementary Material 3: Author positionality and critical reflection**

The authors are academics and practitioners who have designed and implemented research and development programs, together and separately, and have a collective experience spanning public health, engineering, ecology, environmental sciences, architecture, urban planning, international and community development, and experience from 15 countries. Since 2017, we have been designing and implementing the RISE program.

This co-author group intentionally includes voices and experiences from across RISE, at multiple levels.

During the period under study (June 2017 - May 2020), our roles and locations in the RISE program were as follows. Based in Suva, Fiji, Turagabeci was the Fiji Research Lead, Vakarewa was the Fiji Country Manager, and Tella was the Fiji Assessment/Research Team Leader. Turagabeci was involved in the RISE design phase, whereas Vakarewa and Tella commenced in late 2017. In Indonesia, Ansariadi was the Indonesian Research Lead, Ihsan was the Indonesian Intervention Lead, Awaluddin was the Office Manager (late-2017-early 2019) and then the Indonesian Country Manager (2019+), and Taruc was the Assessment/Research Team Leader. Ansariadi and Ihsan were involved in the RISE design phase, whereas Awaluddin and Taruc commenced in late 2017. The named Chief Investigators, which led the original design of RISE, include Brown (P-CI), Ramirez-Lovering, Wong and Leder. All are tenured professors based in Melbourne, Australia. Barker and Henry are mid-career academics, involved in RISE since June 2017. Barker led the day-to-day implementation of Objective 3 and Henry elements of Objective 2. Both have subsequently taken greater leadership roles in RISE as, respectively, Deputy Director, Assessment and Lead for Pathogen and Genomics. French was the RISE Program Manager from June 2017 to September 2021. Davis was the Deputy Program Manager under French, and then became RISE Program Manager. All author institutional affiliations are provided under the paper authorship headings above.

This original idea for the study was conceived by Brown, Leder, Ramirez-Lovering and French. Davis was the Deputy Program Manager under French, and then became RISE Program Manager. French took the lead to develop the study scope, research aims and objectives, and elaborate the proposed methodology. All Members of the RISE Leadership team was presented with the proposed study aims and scope and were invited to be authors. Author contributions, as per the CReditT framework are as follows: French: Conceptualization; Methodology; Investigation; Formal analysis; Writing - Original Draft; Writing - Review & Editing; Visualization; Leder: Conceptualization; Funding acquisition; Methodology; Investigation; Writing - Review & Editing; Vakarewa, Tella, Awaluddin, Taruc, Barker and Henry: Conceptualization; Investigation; Writing - Review & Editing; Turagabeci, Ansariadi, Ihsan: Investigation; Writing - Review & Editing; Ramirez-Lovering and Wong: Funding acquisition; Writing - Review & Editing; Davis: Writing - Review & Editing; Brown: Funding acquisition; Supervision.

We provide the following critical reflections on our roles, positionality and how this may (or may not) have affected the analysis, discussion and conclusions:

- Authorship roles – Writing this paper was driven by the principal author (French), who did most of the analysis and writing. This may have invertedly skewed the analysis towards perspectives anchored in the global north (Australia) and in program management and research administration (French was the program manager). Similarly, as this is an academic journal article, a format that several in our author group do not have extensive experience with, as they are not academics, this may have affected the analysis and presentation of the findings.
- Positionality – The analysis and conclusions should be seen in light of the power dynamics across our author group, including diverse geographies (Australia, Indonesia, Fiji), age and seniority (senior professor, early-career researcher, fieldworkers and lab technicians), expertise (academic, practical, community development), and employment security (tenured academic, fixed-term contract). In essence, this may have affected the analysis by privileging the experiences of the authors that were more experienced in academic research and writing and who had more time available.
- Bias – We are proud of what was accomplished in the first three years and we sought to codify this experience to showcase our efforts. We made it explicit from the outset our desire to be balanced and sensitively explore our experiences from a range of viewpoints. We consciously sought to avoid simple arguments, as well as air our frustrations. These factors may have limited more critical examination of particular actors and experiences.
- Subjectivity – Working closely together for three years, we experienced a range of emotions, from enormous frustration, positive highlights as well as deep disappointments. This experience bonded us as an author team, but potentially limited our ability to be transparently critical of each other’s roles, potentially limiting rigorous and self-critical assessment of our own performance. Similarly, as an author group we spent a lot of time together, in various contexts, leading us to be friends, which may led to ‘group think’, affecting the degree of analysis.
- Representativeness – the author group represents approximately half of the total RISE leadership team. All the leadership team members were invited to co-author, but not all accepted the invitation to participate. Those that declined were mostly the senior researchers (professors) from STEM disciplines. While we are those with more direct day-to-day experience of implementation, this limited the experiences that were drawn from to inform the conclusions.
